# Hemophagocytic Lymphohistiocytosis Mimicking Multisystem Inflammatory Syndrome in Children: A Diagnostic Challenge

**DOI:** 10.7759/cureus.106628

**Published:** 2026-04-08

**Authors:** Mohammad Adawi, Amelia S Mikheev, Allison Bechtel, Thomas Nakagawa, Ayesha Mirza

**Affiliations:** 1 Pediatrics, University of Florida College of Medicine - Jacksonville, Jacksonville, USA; 2 Pediatrics, Washington University School of Medicine, St. Louis Children's Hospital, St. Louis, USA; 3 Pediatric Hematology/Oncology, Nemours Children's Health System, Jacksonville, USA; 4 Pediatric Critical Care Medicine, University of Florida College of Medicine - Jacksonville, Jacksonville, USA; 5 Infectious Diseases, University of Florida College of Medicine - Jacksonville, Jacksonville, USA

**Keywords:** ehrlichia infection, hemophagocytic lymphohistiocytosis (hlh), hyperferritinemia, hyperinflammation, multisystem inflammatory syndrome in children

## Abstract

Hemophagocytic lymphohistiocytosis (HLH) is a life-threatening hyperinflammatory syndrome that can present with clinical and laboratory findings overlapping with multisystem inflammatory syndrome in children (MIS-C). We report a pediatric patient presenting with fever, shock, and elevated inflammatory markers, initially concerning for MIS-C in the setting of prior coronavirus disease exposure. Despite initial management directed toward MIS-C, the patient demonstrated persistent cytopenias and markedly elevated ferritin levels, prompting further evaluation for HLH. Additional workup suggested a potential infectious trigger, including *Ehrlichia *exposure. The patient met diagnostic criteria for secondary HLH and improved following initiation of dexamethasone and doxycycline therapy. This case highlights the diagnostic overlap between hyperinflammatory syndromes in children and emphasizes that persistent cytopenias and extreme hyperferritinemia should prompt early evaluation for HLH, even when MIS-C is initially suspected.

## Introduction

Hyperinflammatory syndromes in children present with overlapping clinical features and can lead to diagnostic delay. Secondary hemophagocytic lymphohistiocytosis (HLH) is a life-threatening hyperinflammatory condition, often infection-triggered, that requires prompt recognition and treatment [1]. Multisystem inflammatory syndrome in children (MIS-C), which emerged during the COVID-19 era, is another important cause of fever, hyperinflammation, and organ dysfunction in pediatric patients [2,3]. Because the clinical and laboratory manifestations of MIS-C overlap with those of other hyperinflammatory conditions, distinguishing MIS-C from alternative diagnoses can be challenging. HLH, particularly when infection-associated, may present with similar findings but requires different management and carries significant morbidity if unrecognized [4].

We describe a child with positive SARS-CoV-2 serology and features initially concerning for MIS-C who was ultimately diagnosed with infection-associated HLH, highlighting the risk of diagnostic anchoring in the post-COVID-19 era and the importance of maintaining a broad differential diagnosis in children presenting with hyperinflammation.

## Case presentation

A previously healthy 10-year-old boy was transferred with seven days of fever, abdominal pain, vomiting, and fatigue. At the referring hospital, he was hypotensive and tachycardic and received fluid resuscitation and empiric broad-spectrum antibiotics prior to transfer. On arrival, he remained tachycardic with borderline hypotension and appeared ill. Examination was notable for cracked lips and right upper quadrant abdominal tenderness without conjunctivitis or lymphadenopathy.

**Figure 1:**
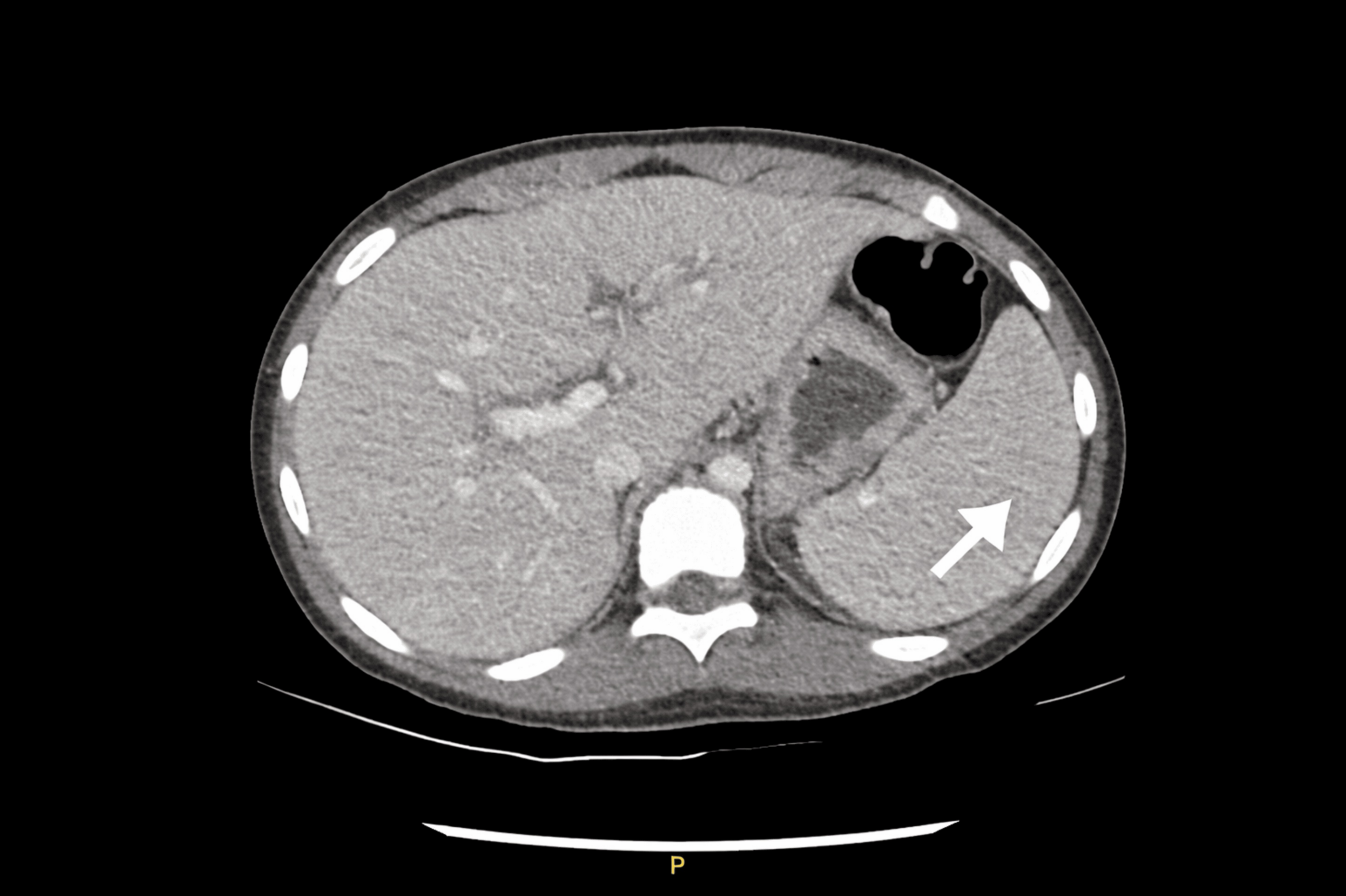
Axial contrast-enhanced CT image of the abdomen showing mild splenomegaly without focal splenic lesions

**Figure 2:**
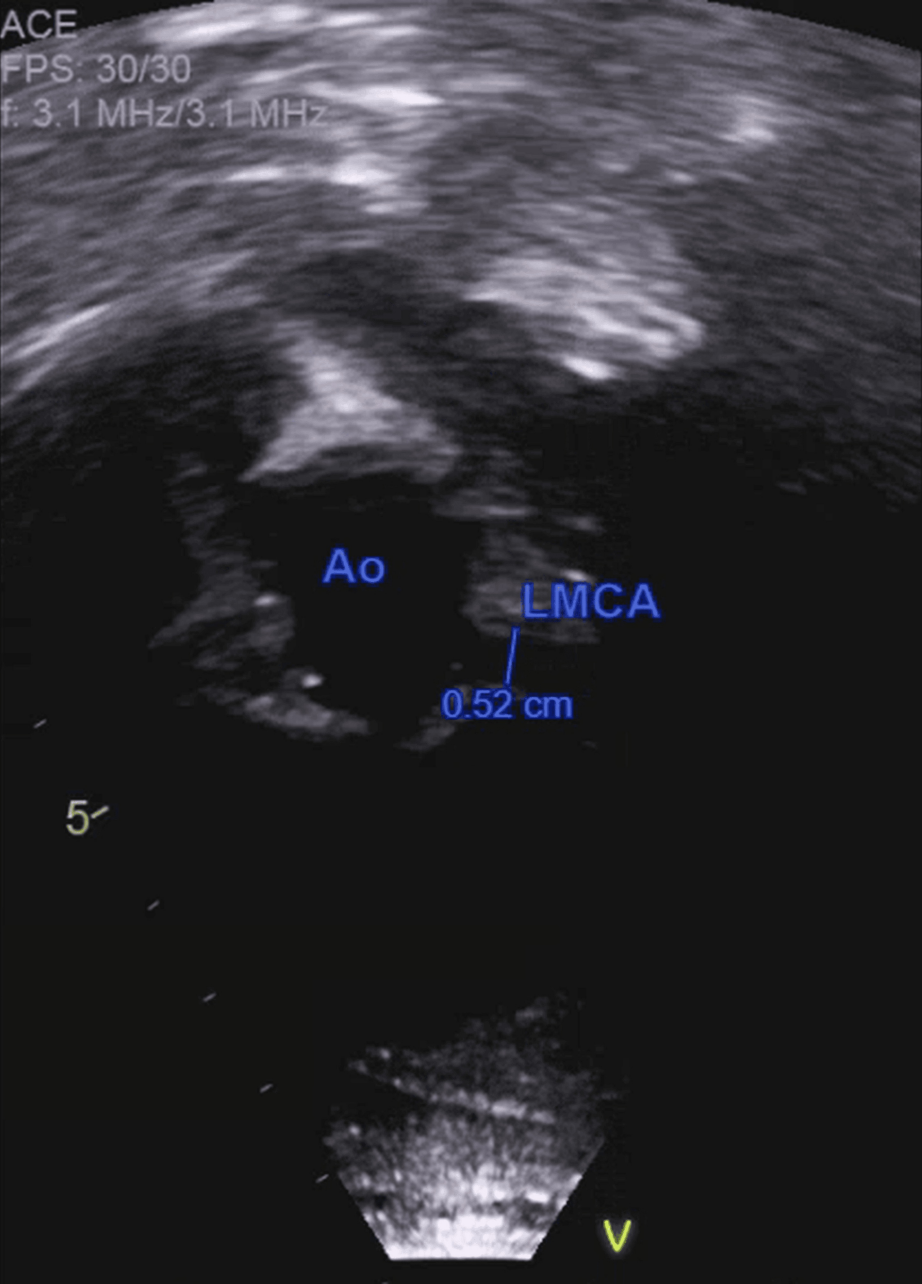
Transthoracic echocardiogram demonstrating dilation of the LMCA origin Parasternal short-axis view at the level of the aortic root showing the LMCA arising from the left coronary sinus. The measured diameter of the LMCA was 0.52 cm, corresponding to a Z-score of 4.2, consistent with coronary dilation. LMCA, left main coronary artery

Laboratory evaluation demonstrated pancytopenia, hyponatremia, hypoalbuminemia, transaminitis, hypertriglyceridemia, elevated lactate dehydrogenase, and markedly elevated ferritin. D-dimer was elevated, and erythrocyte sedimentation rate was normal. SARS-CoV-2 IgG was reactive. Laboratory findings are summarized in Table 1. CT of the abdomen showed mild splenomegaly and small bilateral pleural effusions (Figure 1). Transthoracic echocardiography obtained on admission, prior to initiation of immunomodulatory therapy, revealed diffuse dilation of the left main and proximal left anterior descending coronary arteries (Z-scores 4.2 and 4.1), with borderline left ventricular systolic dysfunction (Figure 2).

**Table 1:** Key laboratory findings at presentation ^*^ IgG reflects prior exposure and does not confirm acute infection. ALT, alanine aminotransferase; AST, aspartate aminotransferase; ESR, erythrocyte sedimentation rate

Test	Patient value	Reference range	Interpretation
White blood cell count (×10³/µL)	2.58	4.5-13.5	Leukopenia
Hemoglobin (g/dL)	8.8	11.2-15.5	Anemia
Platelet count (×10³/µL)	46	150-450	Thrombocytopenia
Serum sodium (mmol/L)	129	135-145	Hyponatremia
AST (U/L)	796	12-47	Transaminitis
ALT (U/L)	303	17-63	Transaminitis
Lactate dehydrogenase (U/L)	1173	130-300	Elevated
Fibrinogen (mg/dL)	141	215-485	Hypofibrinogenemia
Triglycerides (mg/dL)	202	24-150	Hypertriglyceridemia
Ferritin (ng/mL)	14,682	24-336	Markedly elevated
Albumin (g/dL)	2.6	3.4-5.1	Hypoalbuminemia
ESR (mm/hr)	5	3-13	Normal
D-dimer (µg/mL)	5.55	<0.5	Elevated
SARS-CoV-2 IgG	Reactive (qualitative)^*^	-	-

Given shock, hyperinflammation, positive SARS-CoV-2 serology, and coronary artery dilation, MIS-C was initially presumed, and the patient received intravenous immunoglobulin (IVIG) and pulse methylprednisolone.

After 48-72 hours without clinical or laboratory improvement on MIS-C-directed therapy, persistent fever, cytopenias, and markedly elevated ferritin, reaching 24,426 ng/mL, prompted evaluation for HLH. In parallel, an infectious evaluation was initiated to assess alternative diagnoses and potential triggers of secondary HLH. Empiric doxycycline was started while testing was pending because of residence in a tick-endemic region of the southeastern United States. HLH-directed therapy with dexamethasone was initiated, given the overall clinical picture and evolving laboratory abnormalities highly suggestive of HLH.

Subsequent testing demonstrated elevated soluble IL-2 receptor levels, and the patient ultimately fulfilled six of eight HLH-2004 diagnostic criteria (Table 2) [1]. Epstein-Barr virus serology showed positive viral capsid antigen IgM and Epstein-Barr nuclear antigen IgG, consistent with prior infection. Serologic testing for *Ehrlichia chaffeensis* demonstrated elevated IgM and IgG titers. Convalescent serologies and polymerase chain reaction testing were not available; therefore, acute infection could not be definitively confirmed. These findings were interpreted as possible infectious triggers of secondary HLH rather than established primary etiologies. The patient demonstrated rapid clinical and laboratory improvement following initiation of dexamethasone and doxycycline, with resolution of cytopenias and declining ferritin levels.

**Table 2:** HLH-2004 diagnostic criteria and patient findings Total criteria met: six of eight (meeting HLH-2004 diagnostic criteria) HLH, hemophagocytic lymphohistiocytosis; NK, natural killer Based on the HLH-2004 diagnostic criteria described by Henter et al. (2007) [1]

HLH diagnostic criterion	Patient finding	Criterion met
Fever ≥38.5 °C	38.8 °C	✓
Splenomegaly	Present	✓
Cytopenias (≥2 cell lines)	Hemoglobin 8.8 g/dL and platelets 46 × 10³/µL	✓
Hypertriglyceridemia and/or hypofibrinogenemia	Triglycerides 202 mg/dL; fibrinogen 141 mg/dL	✓
Ferritin ≥500 ng/mL	14,682 ng/mL	✓
Hemophagocytosis in bone marrow/spleen/lymph node	Not assessed	✗
Low or absent NK cell activity	Normal activity	✗
Elevated soluble IL-2 receptor	3744 U/mL	✓

Following confirmation of HLH, dexamethasone was continued according to the HLH-94 protocol (etoposide deferred because of clinical stability) [5]. Follow-up echocardiography performed four weeks after treatment initiation demonstrated normalization of coronary artery dimensions and left ventricular function.

## Discussion

During the COVID-19 era, MIS-C became a common consideration in patients presenting with fever, shock, and hyperinflammation. Our patient had several features suggestive of MIS-C, including cardiovascular involvement with coronary artery dilation, systemic inflammation, and reactive SARS-CoV-2 antibodies, which reasonably prompted treatment with IVIG and corticosteroids. Coronary artery dilation is not a typical feature of HLH but has been described in pediatric hyperinflammatory states, including MIS-C, likely reflecting cytokine-mediated endothelial injury rather than disease-specific pathology [6]. However, persistent cytopenias, hypofibrinogenemia, and markedly elevated ferritin were atypical for MIS-C and raised concern for HLH. The patient ultimately fulfilled HLH-2004 diagnostic criteria.

Distinguishing MIS-C from HLH is challenging because both conditions are characterized by fever and multiorgan involvement [7]. Certain laboratory findings may assist differentiation: cytopenias, severe hyperferritinemia, and progressive marrow suppression are more characteristic of HLH, whereas prominent cardiac dysfunction and mucocutaneous findings more commonly support MIS-C [8]. In this case, persistence of cytopenias and extreme ferritin elevation despite MIS-C-directed therapy prompted reassessment of the working diagnosis.

Evaluation for infectious triggers is essential in suspected secondary HLH. Epstein-Barr virus is a well-recognized precipitant [9], and ehrlichiosis has also been reported to trigger HLH [4,10,11]. Our patient demonstrated serologic evidence of EBV exposure and positive* E. chaffeensis* serology. Although acute infection could not be definitively confirmed, these findings were considered potential immune triggers rather than primary etiologies.

This case highlights an important diagnostic pitfall: the tendency to anchor on a diagnosis of MIS-C when children present with hyperinflammation in the post-COVID-19 era. As prior infection and vaccination have become widespread, SARS-CoV-2 serologic positivity may be incidental and should be interpreted cautiously. Failure to reconsider the diagnosis when clinical features are atypical or response to therapy is incomplete may delay appropriate treatment. In children with hyperinflammation, persistent cytopenias, and markedly elevated ferritin, clinicians should broaden the differential diagnosis and evaluate for secondary HLH and infectious triggers, including tick-borne infections.

Early recognition is critical because management and prognosis differ substantially between MIS-C and HLH. While both conditions may initially receive corticosteroids, HLH requires targeted immunosuppression and treatment of an underlying trigger, as well as evaluation for genetic predisposition. Maintaining diagnostic flexibility and reassessing the working diagnosis when the clinical course deviates from expectations are essential to prevent delayed diagnosis and inappropriate therapy.

## Conclusions

In children with suspected MIS-C, persistent cytopenias and markedly elevated ferritin should prompt evaluation for HLH and infectious triggers. Infection-associated HLH may closely mimic MIS-C but requires different management and targeted therapy. Maintaining a broad differential diagnosis is essential to avoid delayed recognition and prevent morbidity.
